# *Aster koraiensis* extract prevents diabetes-induced retinal vascular dysfunction in spontaneously diabetic Torii rats

**DOI:** 10.1186/s12906-017-1998-3

**Published:** 2017-11-23

**Authors:** Junghyun Kim, Kyuhyung Jo, Chan-Sik Kim, Jin Sook Kim

**Affiliations:** 1 Korean Medicine Convergence Research Division, Korea Institute of Oriental Medicine, 1672 Yusengdae-ro, Daejeon, 34054 South Korea; 2 Department of Oral Pathology, School of Dentistry, Chonbuk National University, Jeonju, 54896 South Korea

**Keywords:** *Aster koraiensis*, Blood–retinal barrier, Diabetic retinopathy, Dietary supplement

## Abstract

**Background:**

*Aster koraiensis* extract (AKE) is a standard dietary herbal supplement. The aim of this study is to investigate the inhibitory effects of AKE on diabetes-induced retinal vascular dysfunction in Spontaneously Diabetic Torii (SDT) rats.

**Methods:**

AKE (50 and 100 mg/kg body weight/day) was administered for 16 weeks. The effects of orally administered AKE on blood glucose levels, retinal vascular leakage, apoptosis, and accumulation of advanced glycation end products (AGEs) in the retina were evaluated.

**Results:**

SDT rats exhibited hyperglycemia and retinal vascular leakage, and terminal deoxynucleotidyl transferase dUTP nick end labeling (TUNEL) staining was clearly detected apoptosis in the retinal microvasculature. Immunofluorescence staining revealed the accumulation of AGEs in the retinal vasculature of the SDT rats. However, oral administration of AKE for 16 weeks blocked diabetes-induced blood–retinal barrier (BRB) breakdown and the loss of occludin, which is an important tight junction protein. Apoptosis of retinal vascular cells and AGE accumulation were significantly inhibited after AKE treatment.

**Conclusion:**

These results indicate that, as a dietary herbal supplement, AKE may have beneficial effects on patients with diabetic retinopathy.

## Background

Diabetes-induced retinal dysfunction is a common and serious microvascular complication [1]. Hyperglycemia induces retinal vascular injury and blood–retinal barrier (BRB) breakage, which can cause macular edema in patients with diabetes [2]. The blood vessel endothelium is composed of a single layer of endothelial cells, and the cleft between neighboring endothelial cells is tightly sealed by tight junctions of trans membrane protein complexes such as occludins, claudins, and zona occludens [3]. These complexes contribute to the paracellular barrier, such as the blood–brain barrier and BRB [4]. This endothelial cell barrier can be used as a therapeutic target for vascular permeability-associated diabetic retinopathy [5].

The medical care strategy for patients with diabetic retinopathy is focused on early diagnosis and tight control of blood glucose levels to slow the onset of the disease. Although several classes of glucose-lowering drugs have been developed, the prevalence of diabetic retinopathy is still high [6]. Recently, there has been considerable focus on botanical products that can potentially prevent or treat diabetes and its complications.

*Aster koraiensis* (Korean starwort) is a valuable perennial Korean native plant. This herb is used in food and in folk medicine to treat diseases such as pneumonia, chronic bronchitis, diabetes, and pertussis [7, 8]. In our previous studies, we reported that the extract of *A. koraiensis* inhibits the formation of AGEs and its cross-links with proteins in vitro [9, 10]; and prevented AGE deposition and podocyte apoptosis in the renal tissues of streptozotocin (STZ)-induced diabetic rats [11]. Recently, the extract of *A. koraiensis* was also shown to inhibit retinal pericyte apoptosis in STZ-induced diabetic rats [9]. Although the various effects of *A. koraiensis* extract on diabetes-induced renal and retinal injury in a type 1 diabetes animal model have been reported, the effect on diabetes-induced retinal vascular injury in a type 2 diabetes animal model is unknown. To elucidate this, we investigated the inhibitory effect of *A. koraiensis* extract on the retinal vascular dysfunction in Spontaneously Diabetic Torii (SDT) rats, which is a type 2 diabetes animal model. We also determined the possible mechanism of AKE on the loss of tight junction protein associated with retinal vascular hyperpermeability in this animal model.

## Methods

### Preparation of a. Koraiensis extract

Aerial parts including the flowers, leaves, and stems of *A. koraiensis* were purchased from Gongju, Chungchengnamdo, South Korea in August 2007. The *A. koraiensis* extract (AKE) was prepared according to a previously reported method [11]. Briefly, 2.5 kg of *A. koraiensis* was extracted with EtOH (3 × 20 L) by maceration at room temperature for 3 days. The extracted solution was concentrated to AKE powder (303 g) in vacuum at 40 °C. Voucher specimens of *A. koraiensis* were deposited at the Herbarium of the Korea Institute of Oriental Medicine, Korea (Herbarium No. KIOM-83A). AKE was standardized using high-performance liquid chromatography (HPLC) with, chlorogenic acid and 3, 5-di-*O*-caffeoylquinic acid (Sigma, MO, USA) as reference compounds. The HPLC fingerprint and contents of chlorogenic acid and 3, 5-di-O-caffeoylquinic acid of AKE are described previously [9].

### Animals and experimental design

Male SDT rats and age-matched Sprague-Dawley rats were purchased from CLEA Japan (Tokyo, Japan). At 25 weeks, the rats were randomly divided into four groups of 10 rats, as follows: (1) normal rats (NOR), (2) SDT rats, (3) and (4) SDT rats treated with AKE (SDT + AKE, 50 and 100 mg/kg body weight, respectively). AKE was orally administered for 6 weeks. At necropsy, all rats were sacrificed by CO2 asphyxiation. The animals were housed at the animal facility in Qu-Best Co. Ltd. (Seoul, Korea). All experimental procedures were approved by the Institutional Animal Care and Use Committee at Qu-Best Co. Ltd., Seoul, Korea (IACUC Approval No. QE10072).

### Blood glucose

Blood glucose levels were consistently monitored. Blood samples were collected from the tail vein after a 16 h fast. Blood glucose levels were measured using an automated analyzer (Wako, Tokyo, Japan).

### Fluorescein-dextran microscopy

At the end of the study, rats were anesthetized with isoflurane. Then, 50 mg/mL fluorescein-dextran (Sigma) in phosphate-buffered saline (PBS) was injected into the left ventricle. The tracer dye was allowed to perfuse for 15 min and the eyeballs were then placed in 4% paraformaldehyde for 1.5 h. Blood fluorescein concentration was measured with a fluorometer (Synergy™ HT, Bio-Tek, VT, USA). Dissected retinas were placed on a microscope slide and the whole-mount retinas were observed by fluorescence microscopy (Olympus, Tokyo, Japan). Fluorescence intensity was determined by Image J software (National Institutes of Health, Bethesda, MD, USA) and normalized to the blood fluorescein intensity for each rat.

### Preparation of trypsin-digested vessels

The dissected retinas were fixed in 10% formalin for 24 h. This was followed by incubation in sodium phosphate buffer containing 3% trypsin and 100 mM NaF to inhibit the DNase activity for 1 h. The retinal microvessels were separated from the retinal cells by gentle rinsing in PBS and mounted on slides.

### Terminal deoxynucleotidyl transferase dUTP nick end labeling (TUNEL) staining

The retinal microvessels were stained with a TUNEL fluorescein kit (Promega, Madison, WI, USA) according to the manufacturer’s protocols. TUNEL-positive cells were counted per mm^2^ of capillary area in five fields.

### Immunofluorescence staining

The trypsin digests were hydrated and incubated with either antibodies raised against AGEs (Transgenic Inc., Kobe, Japan) or occludin (Invitrogen, CA, USA) for 1 h at room temperature. Signal detection was achieved using a rhodamine-conjugated goat anti-mouse antibody (Santa Cruz Biotechnology, Paso Robles, CA, USA). As a negative control, tissue sections were incubated with serum from non-immunized animals, instead of the primary antibody. The signal intensity was analyzed in five randomly selected mm^2^ of capillary area from each rat using Image J software.

### Western blot analysis

The retinas were homogenized in (radio-immunoprecipitation assay) RIPA lysis buffer with 1% protease inhibitor cocktail and total protein concentrations were quantified. Protein lysates were separated using SDS–polyacrylamide gel electrophoresis and transferred to polyvinylidene difluoride membranes (Bio-Rad, Hercules, CA, USA). For immunoblotting, the following antibodies were used: rabbit anti-occludin antibody (Invitrogen Life Technologies) and mouse β-actin antibody (Sigma). The immunoreactive bands were visualized and analyzed using an enhanced chemiluminescence detection system (Luminograph II system, Atto Corp., Tokyo, Japan).

### Statistical analysis

Group data were analyzed via one-way analysis of variance followed by Tukey’s multiple comparison test or via unpaired Student’s t-test, using GraphPad Prism 6.0 software (GraphPad, San Diego, CA, USA). Differences with a *p* value <0.05 were considered statistically significant.

## Results

### Blood glucose

Blood glucose levels are summarized in Table 1. The blood glucose levels were significantly high in SDT rats (*p* < 0.05). Blood glucose levels between AKE- and vehicle-treated SDT rats were similar.

**Table 1:** Blood glucose levels

	NOR	SDT	SDT + AKE (mg/kg)	
			50	100
Blood glucose (mg/dL)	164.8 ± 55.15	416.87 ± 71.29*	372.11 ± 93.61	331.25 ± 134.63

All data were expressed as mean ± standard deviation (n = 10)

*p < 0.05 vs. NOR group

### Effect of AKE on diabetes-induced retinal vascular hyperpermeability

**Fig. 1:**
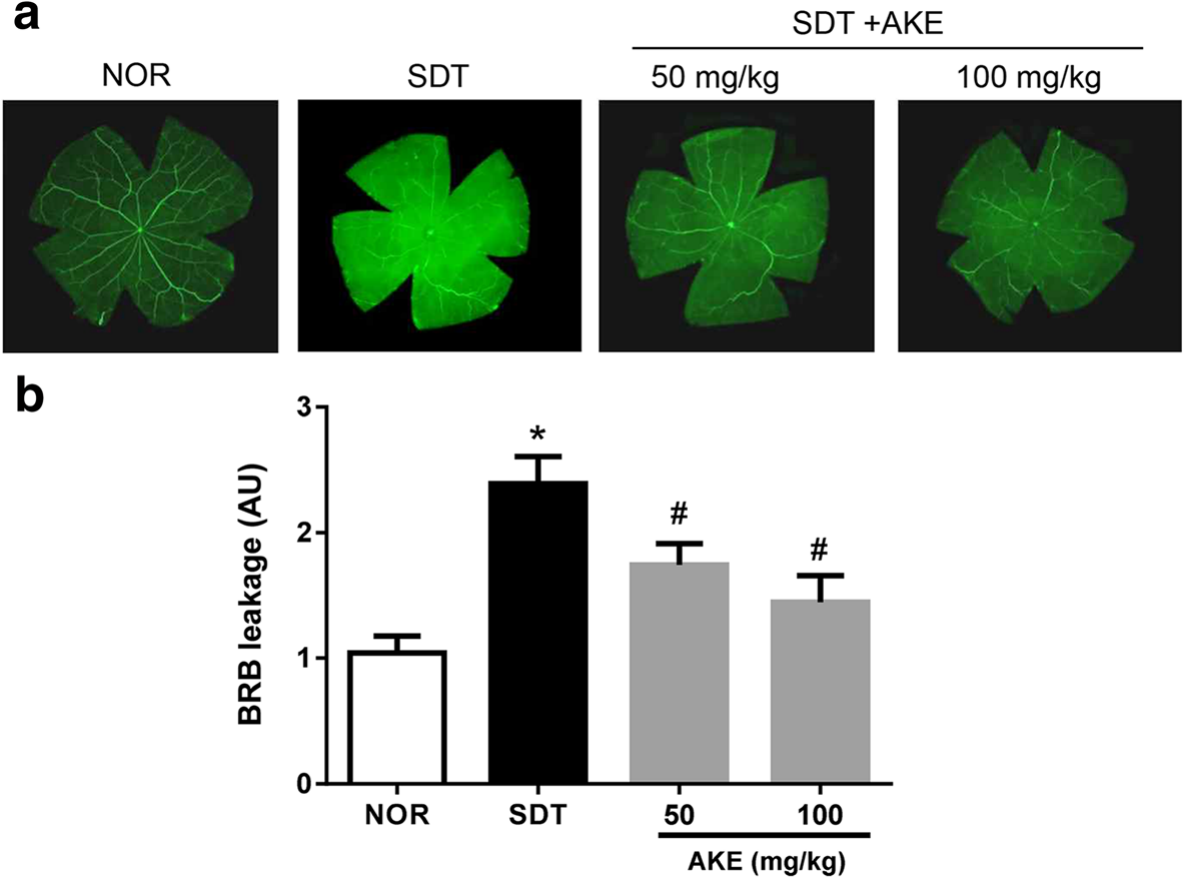
Blood–retinal barrier breakdown. **a** FITC-dextran microscopy on retinal flat mounts. **b** Quantitative analysis of BRB leakage using the FITC-dextran technique. Values in the bar graphs represent the mean ± SE, *n* = 10. *p < 0.05 vs. NOR, #*P* < 0.01 vs. SDT

We determined the retinal vascular leakage using fluorescein-dextran microscopy. In the SDT rats, areas with diffuse fluorescence and reduced delineation of the retinal microvessels were observed. However, AKE-treated SDT rats had smaller areas than those of vehicle-treated SDT rats (Fig. 1a). The quantitative data indicated that AKE treatment prevented the retinal vascular leakage of fluorescein-dextran in a dose-dependent manner (Fig. 1b).

### Effect of AKE on tight junction protein loss in retinal vessels

**Fig. 2:**
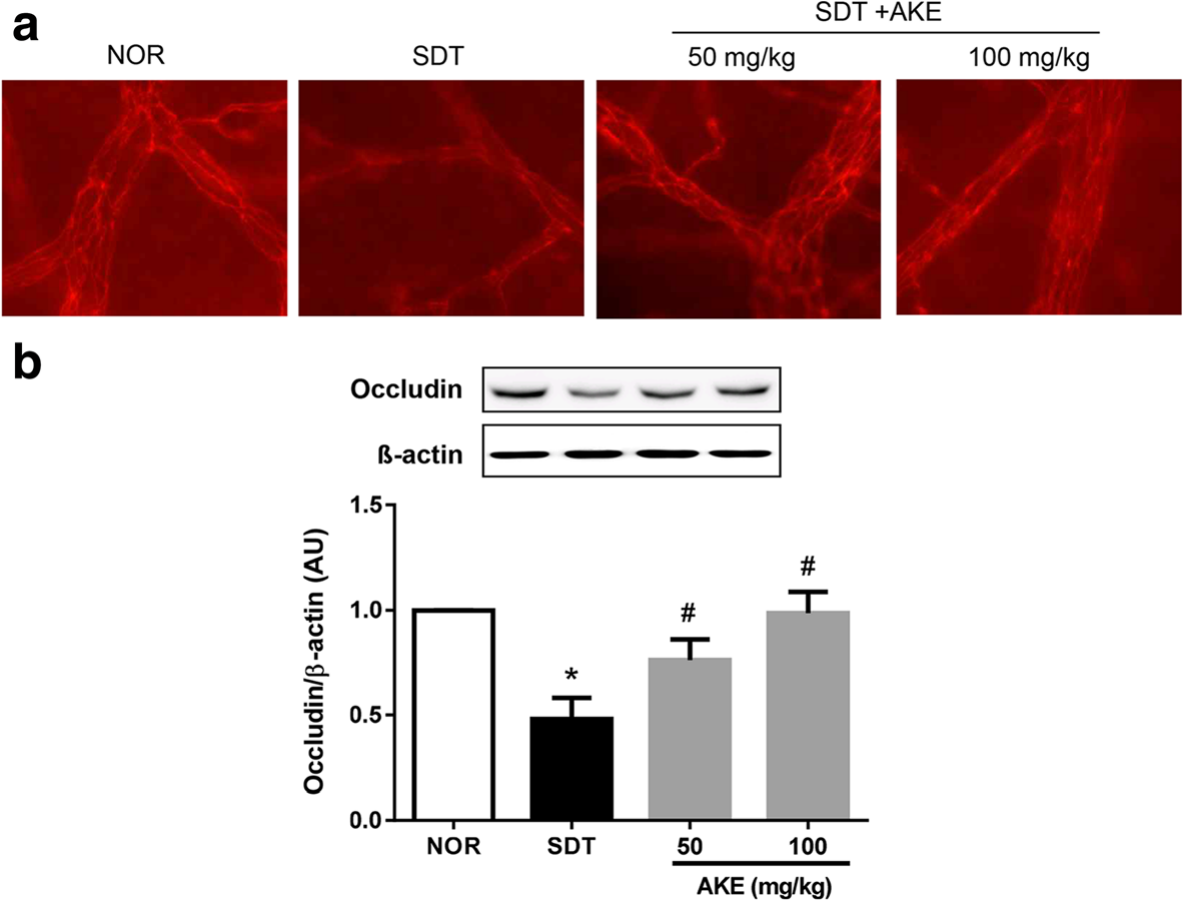
Tight junction protein loss. **a** Immunofluorescence staining for occludin. **b** The total protein was isolated from retinal tissues, and a western blot was performed. Values in the bar graphs represent the mean ± SE, *n* = 5. *p < 0.05 vs. NOR, #*P* < 0.01 vs. SDT

We investigated the expression of occludin in trypsin-digested retinal vessels. As shown in Fig. 2a, occludin in normal control rats was markedly expressed in retinal vessels. However, vehicle-treated SDT rats showed weaker and non-linear occludin expression than that in normal control rats. AKE dose-dependently restored occludin expression in SDT rats. Additionally, a western blot analysis demonstrated that expression levels of occludin significantly reduced in vehicle-treated SDT rats compared with that in normal control rats. However, AKE treatment restored occluding expression in SDT rats in a dose-dependent manner (Fig. 2b).

### Effect of AKE on diabetes-induced retinal vascular apoptosis

**Fig. 3:**
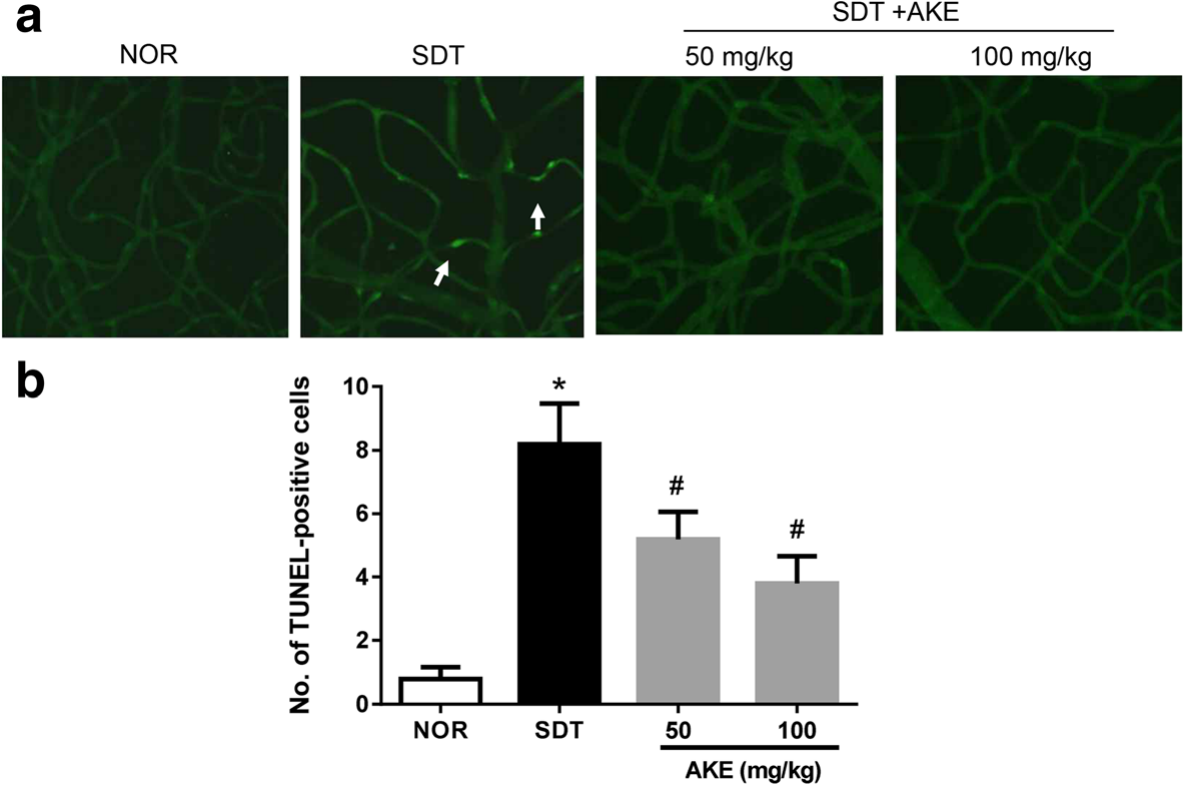
Retinal vascular cell apoptosis. **a** The trypsin-digested retinal vessels were stained with TUNEL. Arrows indicate that apoptotic cell death occurred in the SDT rats. **b** Quantitative analysis of the TUNEL-positive nuclei. Values in the bar graphs represent the mean ± SE, n = 5. *p < 0.05 vs. NOR, #*P* < 0.05 vs. SDT

To identify the injury of retinal vascular cells, we applied the TUNEL assay in trypsin-digested retinal vessels. As shown in Fig. 3a, examination of the retinal trypsin digests of vehicle-treated SDT rats showed several TUNEL-positive cells in the retinal vessel, whereas, relatively few positive cells were observed for normal control and AKE-treated SDT rats. The retinal trypsin digests were analyzed to quantitate TUNEL-positive cells. There were significantly more positive cells in the retinal vessels of vehicle-treated SDT rats than in normal control rats (*p* < 0.05, Fig. 3b). However, AKE reduced the number of positive cells in a dose-dependent manner.

### Effect of AKE on retinal tissue accumulation of AGEs

**Fig. 4:**
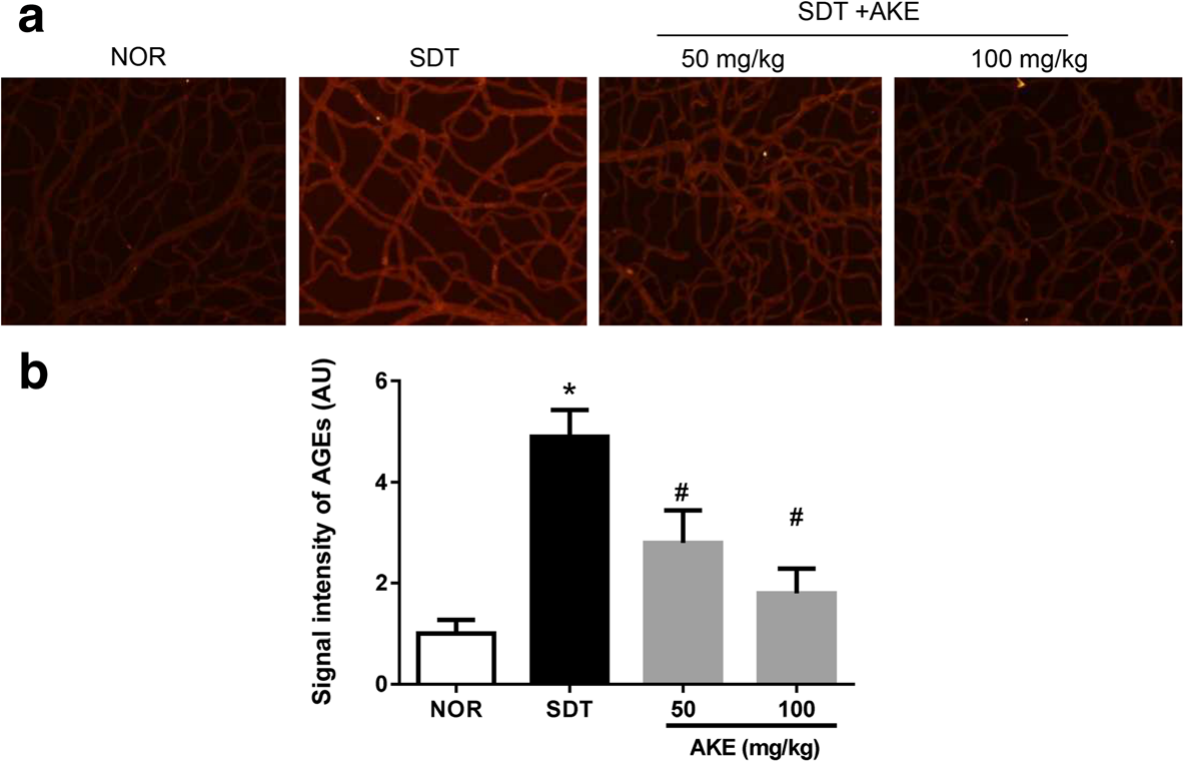
Retinal AGEs accumulations. **a** Immunofluorescence staining for AGEs in trypsin-digested retinal vessels. **b** Quantitative analysis of the AGEs immunoreactive intensities. Values in the bar graphs represent the mean ± SE, n = 5. *p < 0.05 vs. NOR, #*P* < 0.05 vs. SDT

Immunofluorescence staining was performed to evaluate the damage caused by AGEs, which clearly showed accumulation of AGEs in retinal vessels of vehicle-treated SDT rats (Fig. 4a). AGE levels were significantly higher in these animals than that in normal control rats. However, SDT rats treated with AKE showed a dose-dependent reduction in retinal accumulation of AGEs (Fig. 4b).

## Discussion

Currently, several dietary herbal supplements with beneficial effects on health are sold worldwide. In this study, we showed that AKE, as a dietary herbal supplement, ameliorates diabetes-induced retinal vascular dysfunction in a type 2 diabetes animal model. AKE-treated SDT rats showed significant improvements in retinal vascular function markers, such as vascular permeability. Additionally, AKE reduced apoptosis and AGE accumulation in retinal vascular cells.

In our previous study, we reported that chlorogenic acid is a bioactive compound of AKE and that the content of chlorogenic acid in AKE is 1.24%. An oral dose of 100 mg/kg AKE contained 1.24 mg/kg chlorogenic acid is recommended [9]. Qi et al. reported that the peak plasma concentration of chlorogenic acid after oral administration of 50 mg/kg chlorogenic acid to rats was 550 μg/ml [12]. Although we used a relatively low dose of chlorogenic acid, a concentration of 52.55 μg/ml chlorogenic acid is sufficient to inhibit AGE formation in vitro [9]. Indeed, an oral administration of AKE (100 mg/kg body weight) in STZ-induced diabetic rats reduced the AGEs deposited in the retinal microvascular cells. Thus, we chose the oral doses of 50 and 100 mg/kg AKE to evaluate their inhibitory effects on retinal AGEs accumulations in the SDT rats.

The SDT rat is a newly developed non-obese type 2 diabetes animal model that spontaneously develops hyperglycemia resulting from reduced insulin secretion [13]. An SDT rat is the most appropriate animal model to investigate diabetic retinopathy. Retinal vascular leakage, vascular cell loss, and proliferative neovascularization are characteristics of SDT rats, thereby resembling the clinical features of human diabetic retinopathy [14, 15].

The BRB is critical for the maintenance of normal microvascular function, and its breakdown is an early clinical feature of diabetic retinopathy [16]. The retinal vascular endothelium has high trans-endothelial resistance due to intercellular tight junctions [17]. Occludin was the first identified component of tight junction strands and has an important role in the regulation of endothelium electrical resistance, permeability, and barrier function [18]. In this study, fluorescein-dextran microscopy demonstrated that AKE significantly decreased fluorescein leakage, suggesting that AKE might prevent BRB breakdown. Moreover, the loss of occludin was markedly inhibited after the administration of AKE. These results suggest that AKE can prevent retinal vascular leakage under diabetic conditions.

In the retinal tissues, extensive formation and accumulation of AGEs is considered responsible for the pathogenesis of diabetic retinopathy [19, 20]. Repeated intravenous (i.v.) injection of exogenous AGEs into animals with normal blood glucose levels resulted in BRB breakdown in the retinas [21]. It has been reported that AGEs elicit pro-apoptotic cytokine release or reactive oxygen species (ROS) generation through the AGE/RAGE (receptor for AGE) interaction, resulting in apoptotic cell death of retinal vascular cells [22–24]. Moreover, anti-AGE agent, aminoguanidine, prevented the diabetes-induced retinal vascular injury [25]. In our previous study, we reported that AKE has a potent anti-AGE activity. In STZ-induced diabetic rats, a type 1 diabetes animal model, oral administration of AKE (100 mg/kg body weight) for 4 months reduced the AGEs deposited in the retinal microvascular cells [9]. Similarly, the present study showed that AKE (50 and 100 mg/kg body weight) also dose-dependently prevented AGE accumulation and vascular cell apoptosis in the retinal vessels of SDT rats. Based on these findings, we suggest that AKE potentially ameliorates diabetic retinopathy through anti-AGE activity and that the minimum effective dose of AKE was 50 mg/kg body weight.

Chlorogenic acid and 3, 5-di-*O*-caffeoylquinic acid are two major compounds present in AKE [9]. Kim et al. showed that chlorogenic acid could inhibit the formation of AGEs and the AGE/RAGE interaction in vitro [9, 10]. Chlorogenic acid has a chelating activity towards methylglyoxal [26], which can disturb the formation of AGEs. 3, 5-Di-*O*-caffeoylquinic acid prevented the formation of AGEs with approximately 20-fold higher activity than chlorogenic acid [9]. These findings suggest that the preventive effects of AKE on diabetic retinopathy may be ascribed to the synergistic effects of these two active components.

## Conclusion

AKE ameliorated diabetes-induced retinal vascular dysfunction in SDT rats. We also demonstrated that AKE protected these diabetic animals from AGE-related retinal vascular injury. Our data suggest that the oral administration of AKE as a dietary supplement can potentially benefit patients with diabetic retinopathy.

## Abbreviations

AGEs: Advanced glycation end products
AKE: *Aster koraiensis* extract
BRB: Blood–retinal barrier
HPLC: High-performance liquid chromatography
NOR: Normal rats
PBS: Phosphate-buffered saline
SDT: Spontaneous diabetic Torii
STZ: Streptozotocin
TUNEL: Terminal deoxynucleotidyl transferase dUTP nick end labeling

## References

[1] Fong DS, Sharza M, Chen W, Paschal JF, Ariyasu RG, Lee PP (2002). Vision loss among diabetics in a group model health maintenance organization (HMO). Am J Ophthalmol.

[2] Ciulla TA, Amador AG, Zinman B (2003). Diabetic retinopathy and diabetic macular edema: pathophysiology, screening, and novel therapies. Diabetes Care.

[3] Mehta D, Malik AB (2006). Signaling mechanisms regulating endothelial permeability. Physioligical Rev.

[4] Harhaj NS, Antonetti DA (2004). Regulation of tight junctions and loss of barrier function in pathophysiology. Int J Biochem Cell Biol.

[5] Barber AJ (2003). A new view of diabetic retinopathy: a neurodegenerative disease of the eye. Prog Neuro Psychopharmacol Biol Psychiat.

[6] Girach A, Manner D, Porta M (2006). Diabetic microvascular complications: can patients at risk be identified? A review. Int J Clin Pract.

[7] Ahn DK (1998). Illustrated book of Korean medicinal herbs.

[8] Ko JY, Lee KK (1996). Effect of plant growth regulators on growth and flowering of potted *Lychnis cognata, Aster koraiensis*, and *Campanula takesimana*. RDA J Agric Sci.

[9] Kim J, Jo K, Lee IS, Kim CS, Kim JS. The extract of Aster Koraiensis prevents retinal Pericyte apoptosis in diabetic rats and its active compound, Chlorogenic acid inhibits AGE formation and AGE/RAGE interaction. Nutrients. 2016;8(9):585. 10.3390/nu8090585 PMC5037569 27657123

[10] Lee J, Lee YM, Lee BW, Kim JH, Kim JS (2012). Chemical constituents from the aerial parts of Aster Koraiensis with protein glycation and aldose reductase inhibitory activities. J Nat Prod.

[11] Sohn E, Kim J, Kim CS, Kim YS, Jang DS, Kim JS (2010). Extract of the aerial parts of Aster Koraiensis reduced development of diabetic nephropathy via anti-apoptosis of podocytes in streptozotocin-induced diabetic rats. Biochem Biophys Re Commun.

[12] Qi W, Zhao T, Yang W-W, Wang G-H, Yu H, Zhao H-X, Yang C, Sun L-X (2011). Comparative pharmacokinetics of chlorogenic acid after oral administration in rats. J Pharml Anal.

[13] Sasase T, Ohta T, Ogawa N, Miyajima K, Ito M, Yamamoto H, Morinaga H, Matsushita M (2006). Preventive effects of glycaemic control on ocular complications of spontaneously diabetic Torii rat. Diab Obes Metab.

[14] Masuyama T, Komeda K, Hara A, Noda M, Shinohara M, Oikawa T, Kanazawa Y, Taniguchi K (2004). Chronological characterization of diabetes development in male spontaneously diabetic Torii rats. Biochem Biophys Res Commun.

[15] Shinohara M, Masuyama T, Shoda T, Takahashi T, Katsuda Y, Komeda K, Kuroki M, Kakehashi A, Kanazawa Y (2000). A new spontaneously diabetic non-obese Torii rat strain with severe ocular complications. Int J Exp Diabetes Res.

[16] Navaratna D, McGuire PG, Menicucci G, Das A (2007). Proteolytic degradation of VE-cadherin alters the blood-retinal barrier in diabetes. Diabetes.

[17] Watson PM, Anderson JM, Vanltallie CM, Doctrow SR (1991). The tight-junction-specific protein ZO-1 is a component of the human and rat blood-brain barriers. Neurosci Lett.

[18] Hawkins BT, Davis TP (2005). The blood-brain barrier/neurovascular unit in health and disease. Pharmacol Rev.

[19] Koga K, Yamagishi S, Okamoto T, Inagaki Y, Amano S, Takeuchi M, Makita Z (2002). Serum levels of glucose-derived advanced glycation end products are associated with the severity of diabetic retinopathy in type 2 diabetic patients without renal dysfunction. Int J Clin Pharmacol Res.

[20] Miura J, Yamagishi S, Uchigata Y, Takeuchi M, Yamamoto H, Makita Z, Iwamoto Y (2003). Serum levels of non-carboxymethyllysine advanced glycation endproducts are correlated to severity of microvascular complications in patients with type 1 diabetes. J Diabetes Complicat.

[21] Stitt AW, Bhaduri T, McMullen CB, Gardiner TA, Archer DB (2000). Advanced glycation end products induce blood-retinal barrier dysfunction in normoglycemic rats. Mol Cell Biol Res Commun : MCBRC.

[22] Kasper M, Roehlecke C, Witt M, Fehrenbach H, Hofer A, Miyata T, Weigert C, Funk RH, Schleicher ED (2000). Induction of apoptosis by glyoxal in human embryonic lung epithelial cell line L132. Am J Respir Cell Mol Biol.

[23] Kaji Y, Amano S, Usui T, Oshika T, Yamashiro K, Ishida S, Suzuki K, Tanaka S, Adamis AP, Nagai R (2003). Expression and function of receptors for advanced glycation end products in bovine corneal endothelial cells. Investig Ophthalmol Vis Sci.

[24] Yamagishi S, Inagaki Y, Amano S, Okamoto T, Takeuchi M, Makita Z (2002). Pigment epithelium-derived factor protects cultured retinal pericytes from advanced glycation end product-induced injury through its antioxidative properties. Biochem Biophys Res Commun.

[25] Kern TS, Engerman RL (2001). Pharmacological inhibition of diabetic retinopathy: aminoguanidine and aspirin. Diabetes.

[26] Kim J, Jeong IH, Kim CS, Lee YM, Kim JM, Kim JS (2011). Chlorogenic acid inhibits the formation of advanced glycation end products and associated protein cross-linking. Arch Pharm Res.

